# Systemic Inflammatory Response Index and Futile Recanalization in Patients with Ischemic Stroke Undergoing Endovascular Treatment

**DOI:** 10.3390/brainsci11091164

**Published:** 2021-08-31

**Authors:** Simona Lattanzi, Davide Norata, Afshin A. Divani, Mario Di Napoli, Serena Broggi, Chiara Rocchi, Santiago Ortega-Gutierrez, Gelsomina Mansueto, Mauro Silvestrini

**Affiliations:** 1 Neurological Clinic, Department of Experimental and Clinical Medicine, Marche Polytechnic University, 60020 Ancona, Italy; dav.norata@gmail.com (D.N.); serena.broggi@gmail.com (S.B.); chiararocchi@hotmail.it (C.R.); m.silvestrini@univpm.it (M.S.); 2 Department of Neurology, University of New Mexico, Albuquerque, NM 87131, USA; adivani@gmail.com; 3 Neurological Service, SS Annunziata Hospital, Sulmona, 67039 L’Aquila, Italy; mariodinapoli@katamail.com; 4 Departments of Neurology, Neurosurgery, and Radiology, University of Iowa, Iowa City, IA 52242, USA; santy-ortega@uiowa.edu; 5 Department of Advanced Medical and Surgical Sciences, University of Campania “Luigi Vanvitelli”, 80138 Naples, Italy; gelsomina.mansueto@unicampania.it; 6 Clinical Department of Laboratory Services and Public Health—Legal Medicine Unit, University of Campania “Luigi Vanvitelli”, 80138 Naples, Italy

**Keywords:** cerebrovascular disease, endovascular treatment, intravenous thrombolysis, inflammation, ischemic stroke, lymphocyte, mechanical thrombectomy, monocytes, neutrophil, recanalization, systemic inflammation response index

## Abstract

Futile recanalization remains a significant challenge for endovascular treatment (EVT) of acute ischemic stroke (AIS). The inflammatory response that occurs after cerebral infarct plays a central role in stroke pathobiology that can influence the outcome of a recanalization procedure. The aim of this study was to evaluate the relationship between the systemic inflammatory response index (SIRI) and futile recanalization in patients with AIS. We retrospectively identified consecutive patients with ischemic stroke due to proximal arterial occlusion in the anterior circulation, who were treated with EVT and achieved near-complete or complete recanalization. Absolute neutrophil count (ANC), absolute monocyte count (AMC), and absolute lymphocyte count (ALC) were collected from admission blood work to calculate SIRI as ANC × AMC/ALC. The study outcome was futile recanalization, defined as poor functional status [modified Rankin scale (mRS) score ≥ 3] at 3 months despite complete or near-complete recanalization. A total of 184 patients were included. Futile recanalization was observed in 110 (59.8%) patients. Older patients (odds ratio (OR) = 1.07, 95% confidence interval (CI): 1.04–1.10, *p* < 0.001), higher admission National Institutes of Health stroke scale score (OR = 1.10, 95% CI: 1.02–1.19, *p* = 0.013), and higher admission SIRI (OR = 1.08, 95% CI: 1.01–1.17, *p* = 0.028) increased the risk of the poor outcome at 3 months despite complete or near-complete recanalization.

## 1. Introduction

Stroke is a leading cause of morbidity and mortality and a major determinant of health expenditures worldwide. Recent advances in stroke treatment have significantly reduced the overall mortality rate, whereas the number of survivors with complications and disability is still high [1,2,3]. Of note, the ability to recanalize occluded intracranial vessels has improved with the introduction of new devices. However, futile recanalization, which consists in the lack of the achievement of a good clinical outcome despite the achievement of a successful recanalization of the occluded vessel, remains a significant challenge for endovascular treatment (EVT) of acute ischemic stroke (AIS). Indeed, while recanalization is reached in more than three quarters of patients, the rate of associated favorable outcomes remains below 50%, suggesting that futile recanalization is a relatively common phenomenon [4]. Predicting futile recanalization early after index stroke represents an important research question for improving the outcome of these patients.

Systemic and local inflammatory responses represent key steps in determining tissue damage in almost every tissue after several and different pathogenic stimuli [5,6]. These responses also play a key role in the pathophysiology of ischemic stroke, being implicated in the secondary progression of ischemic lesions as well as infarct resolution, remodeling, and tissue repair [7]. Several studies have explored and identified the overall association between serum inflammatory biomarkers and outcome in patients with ischemic stroke [8,9,10,11]. Conversely, there is limited evidence about the relationship between inflammatory biomarkers and functional recovery in patients with AIS who achieved successful recanalization. In this perspective, the systemic inflammatory response index (SIRI), obtained from the neutrophils, monocytes, and lymphocytes counts, has been recently reported as an easily available and inexpensive synthesis of the immune pathways and measure of the inflammatory levels. In recent years, the SIRI has been mostly studied as a predictor of outcome in patients affected by tumors, whereas its utility in the field of cerebrovascular diseases has been only marginally explored, being so far associated with the risk of ischemic stroke in patients with rheumatoid arthritis [12] and the prognosis of patients with aneurysmal subarachnoid hemorrhage [13].

This study aimed to evaluate the relationship between the SIRI and 3-month functional outcome in patients with AIS who underwent a successful EVT recanalization.

## 2. Methods

### 2.1. Study Participants 

We retrospectively identified consecutive patients with AIS who were admitted at the Stroke Unit of the Marche Polytechnic University (Ancona, Italy) from January 2016 to October 2019 and were treated with intravenous thrombolysis (IVT) plus EVT or EVT alone. Patients were included if they had intracranial proximal arterial occlusion in the anterior circulation (i.e., intracranial carotid artery or M1/M2 segments of middle cerebral artery) demonstrated by vascular imaging such as computed tomographic angiography, magnetic resonance angiography, or digital subtraction angiography, received IVT within 4.5 h and started EVT within 6.0 h after the onset of stroke, and achieved near-complete or complete recanalization (TICI 2b or 3) [14]. Intravenous thrombolysis consisted of the administration of recombinant tissue plasminogen activator (rt-PA) at the dose of 0.9 mg/kg (maximum 90 mg; 10% bolus followed by a 60 min infusion). EVT consisted of mechanical thrombectomy with aspiration catheters alone, stent-retrievers alone, or both, depending on occlusion type/location and neuro-interventionist’s choice. Demographic data, vascular risk factors, medical history, and baseline stroke severity according to the National Institutes of Health Stroke Scale (NIHSS) score [15] were retrieved from medical records, as previously detailed [16,17,18]. The ischemic lesion extension was estimated according to the Alberta Stroke Program Early CT Score (ASPECTS) on head computed tomography performed at the Emergency Department [19]. Total white blood cells, absolute neutrophil count (ANC), absolute monocyte count (AMC), and absolute lymphocyte count (ALC) were collected from admission blood work within 24 h after stroke onset. The SIRI was computed as follows: SIRI = ANC × AMC/ALC [13]. The outcome measure was futile recanalization, defined as poor 3-month functional status (modified Rankin Scale (mRS) score ≥3) [20] despite complete or near-complete recanalization [21]. Patients with a pre-stroke mRS score >2 and patients who did not have admission laboratory values available and/or 3-month outcome assessed by direct clinical evaluation as part of routine clinical follow-up were excluded.

### 2.2. Statistical Analysis 

Values were presented as mean ± standard deviation (SD) or median (interquartile range [IQR]) for continuous variables and as the number (%) of subjects for categorical variables. Univariate comparisons were made through the student *t*-test, Mann–Whitney test, or chi-squared test, as appropriate. The association between the SIRI and study outcome was determined using logistic regression. The variables with *p*-values <0.05 from the comparison of baseline characteristics and associations with biologically plausible outcomes were forced into the multivariate analysis; selected variables were age, sex, initial NIHSS score, and ASPECT score [21]. The receiver operating characteristic (ROC) analysis was performed to evaluate the ability of the SIRI to predict the futile recanalization. The cut-off point that better distinguished the presence and absence of the study outcome was determined as the value with the highest Youden’s index [22]. The collinearity between exposure variables was assessed with the variance inflation index. Results were considered significant for *p*-values <0.05 (two-sided). Data analysis was performed using STATA/IC 13.1 statistical package (StataCorp LP, College Station, TX, USA).

## 3. Results

**Figure 1:**
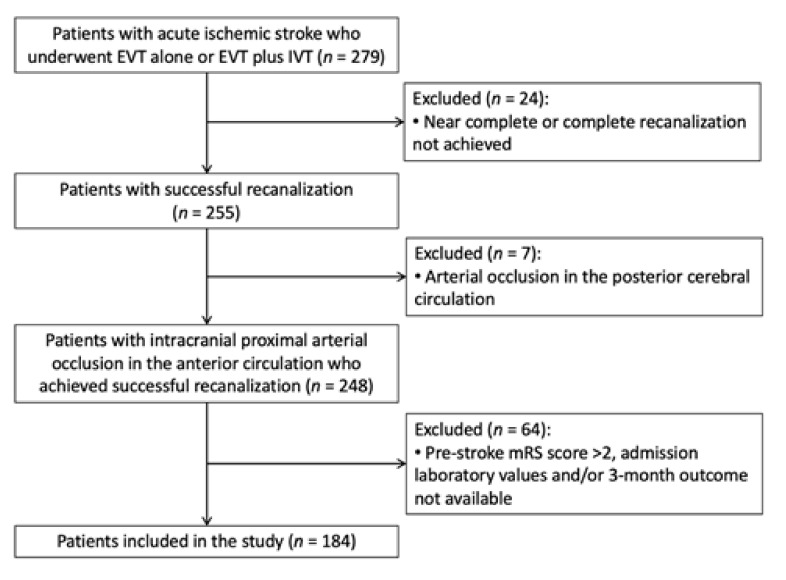
Patient selection flow diagram. Abbreviations: EVT—endovascular treatment, IVT—intravenous thrombolysis, mRS—modified Ranking scale.

A total of 255 out of 279 ischemic stroke patients undergoing treatment with EVT alone or EVT plus IVT achieved successful recanalization of occluded vessels, defined as near-complete or complete recanalization. Seven patients were excluded due to occlusion of posterior brain circulation, and 64 patients were excluded due to pre-stroke mRS >2 or unavailability of admission laboratory values and/or 3-month outcome. Accordingly, 184 patients were included in the analysis (Figure 1).

The median age of the patients was 75 (61–81) years, and 87 (47.3%) were males. Futile recanalization was observed in 110 out of 184 (59.8%) patients. The futile recanalization group was older (79 (69–83) versus 64 (53–76) years; *p* < 0.001), had a higher prevalence of hypertension (71.8 versus 51.4%, *p* = 0.005), had a higher baseline NIHSS score (16 (13–18) versus 15 (10–18), *p* = 0.011), had a lower admission lymphocyte count (1130 (750–1580) × 10^9^/L versus 1325 (970–1970) × 10^9^/L, *p* = 0.012), and a higher SIRI at admission (4.6 (2.4–8.3) × 10^9^/L versus 3.3 (1.6–5.3) × 10^9^/L, *p* < 0.001) compared with patients without futile recanalization. Baseline demographics and clinical characteristics of the study cohort according to the incidence of futile recanalization are shown in Table 1.

Age, baseline NIHSS score, and admission SIRI were independent predictors of futile recanalization: patients with older age (odds ratio (OR) = 1.07, 95% confidence interval (CI): 1.04–1.10, *p* < 0.001), higher NIHSS score (OR = 1.10, 95% CI: 1.02–1.19, *p* = 0.013), and higher SIRI (OR = 1.08, 95% CI: 1.01–1.17, *p* = 0.028) had an increased risk of poor 3-month outcome despite complete or near-complete recanalization (Table 2). The multivariate model did not suffer from collinearity (variance inflation factors ranged from 1.03 to 1.29). The SIRI outperformed admission neutrophil, monocyte, and lymphocyte counts in the prediction of futile recanalization (data not shown).

**Figure 2:**
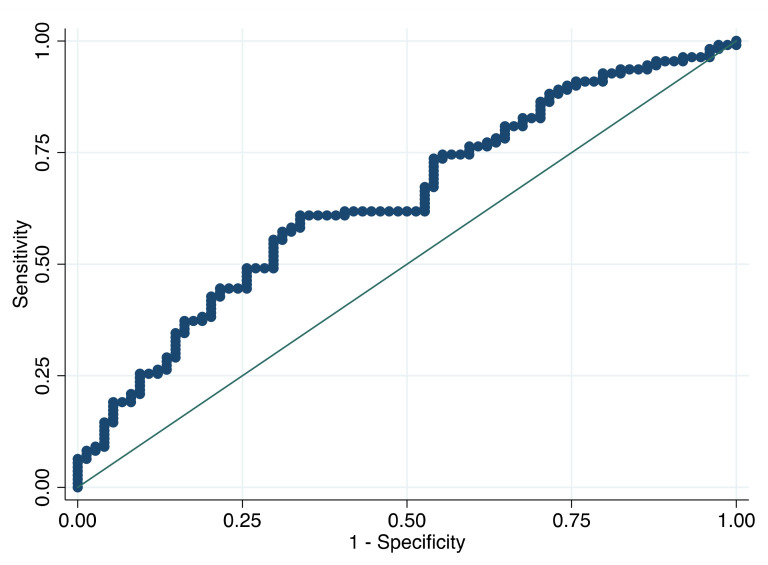
Receiver operating characteristic curve for the prediction of futile recanalization. Predictive values of the SIRI for futile recanalization. Area under the curve 0.644 (95% CI: 0.563–0.725). Abbreviations: CI—confidence interval, SIRI—systemic inflammation response index.

For the ROC analysis with respect to the outcome of futile recanalization, the AUC was 0.644 (95% CI: 0.563–0.725) with 3.8 × 10^9^/L as the best predictive cut-off value of SIRI (Figure 2). Futile recanalization occurred in 72.0% and 47.3% of the patients with SIRI ≥ 3.8 × 10^9^/L and <3.8 × 10^9^/L, respectively (*p* = 0.001). The SIRI ≥3.8 × 10^9^/L was an independent predictor of futile recanalization (OR = 2.88, 95% CI: 1.56–5.30), *p* < 0.001 and adjusted OR = 2.11, 95% CI: 1.07–4.19, *p* = 0.032).

## 4. Discussion

The main novel finding of this study is the association between SIRI and futile recanalization in patients with AIS. Patients with ischemic stroke undergoing EVT and reaching successful recanalization who had higher SIRI at admission were at increased risk of poor 3-month functional outcome. The SIRI was a readily available and independent predictor of futile recanalization, and the likelihood of unfavorable 3-month status was increased nearly two-fold in patients with SIRI above the identified threshold of 3.8 × 10^9^/L. These results are consistent with the growing evidence that inflammation plays a central role in stroke pathobiology and can influence the outcome [23]

The inflammatory response occurs soon after stroke following the release of inflammatory mediators from damaged brain tissue. Microglia, which are brain resident macrophages, are activated, and circulating immune cells are recruited to the injury site [24]. The prominent influx of polymorphonuclear cells and monocytes, which give rise to macrophages, into the brain is one of the earliest events in the inflammatory cascade. In the acute phase, infiltrated leukocytes produce inflammatory cytotoxic mediators that promote cellular injury, increase capillary permeability, and trigger pro-thrombotic pathways leading to exacerbation of ischemic injury, edema development, and secondary progression of tissue damage [25,26]. While the ischemic core tissue cannot be salvaged, the penumbra region around the ischemic core can be salvaged if blood flow is restored in a timely fashion. In this regard, microvascular compromise due to plugging of micro-vessels that follows endothelial activation and is mediated by leukocyte recruitment and platelet aggregation can influence the actual tissue reperfusion at the capillary level and, hence, impair the viability of the penumbra area despite recanalization of the large vessels [21,27]. Experimental evidence has shown that blood flow is not restored in some randomly distributed brain areas after reopening of the cerebral circulation [28], and platelets and leukocytes contribute to the ‘no-reflow’ phenomenon [29]. The recanalization and reperfusion of the previously hypoxic brain areas increase the pro-inflammatory function of platelets and activate complex thrombo-inflammatory pathways that contribute to the ischemia–reperfusion injury [29]. Of note, T cells interact with platelets and facilitate further infarct development and increase of infarct size. In this regard, microvascular events and secondary thrombosis in the microvasculature involve endothelium–leukocyte–platelet interactions and can account for the ongoing infarct growth despite recanalization [29].

As the overall regenerative capacities of neural cells in the chronic phase are limited, the severity of these early events and secondary-induced brain damage and cellular loss can significantly influence stroke recovery and impair the outcome [30].

The acute injury to the brain can also increase catecholamine and cortisol levels that, in turn, induce the apoptosis and functional deactivation of peripheral lymphocytes [31]. Being key regulators of the cellular and humoral responses, the loss of lymphocytes can result in impaired host defense against pathogens and increased vulnerability to infections. Of note, infections are among the most common complications after stroke and may worsen the clinical course [32,33]. In addition, specific subpopulations of regulatory lymphocytes preserve the immune homeostasis and act as neuroprotective modulators of the immune response by counteracting the production of pro-inflammatory mediators, modulating the microglia activation, containing the autoreactive cells, and promoting the neurogenesis and repair processes in the ischemic region [34,35,36].

Our data expand the currently available evidence about the role of plasma biomarkers in predicting stroke outcome. In this context, it is worth emphasizing that the assessment of one single cellular line may not be enough to capture the complexity of the immune status and response. Further, a single blood cell test can be affected and biased by conditions such as overhydration, dehydration, and handling of blood specimens. Accordingly, indices and ratios that incorporate multiple cell measurements may be more reliable measures to use in clinical practice [37,38,39]. The SIRI was directly and independently related to the risk of futile recanalization and can be a useful complement to clinical predictors, such as age and baseline stroke severity that have been already reported in the literature [4,40].

The SIRI reflects the balance between innate and adaptive immunity, with higher values synthetizing the increased activity of the former and the decreased of the latter. In the acute phase of cerebral infarct, the SIRI can represent the hyper-acute inflammatory response to brain injury and integrate the likelihood of secondary damage and the susceptibility to post-stroke complications.

Recently, increased levels of matrix-metalloproteinase-9, tenascin-C, and thioredoxin, and decreased levels of a disintegrin and metalloproteinase with a thrombospondin motif repeats 13 (ADAMTS13) and gelsolin have been identified as independent predictors of unfavorable outcome in ischemic stroke patients after successful recanalization by EVT [41]. Compared to these biomarkers, the SIRI has the advantage to be a readily available, cost-effective, and easily accessible index as derived from widely accessible laboratory variables usually collected in routine medical practice. The real-world design, which ensures the generalizability of the findings to everyday clinical settings, and the exclusion of patients with unsuccessful vessel recanalization after EVT, which assures greater homogeneity of the enrolled cohort, may represent additional study strengths. Nonetheless, different shortcomings need to be considered. The retrospective data collection at a single academic center could have resulted in selection bias, and the small sample size prevented subgroup analyses. Only a limited set of variables was considered, and the SIRI was estimated through peripheral blood cells counts obtained at one single time point rather than over time through repeated measurements, preventing the exploration of the dynamic evolution of the inflammatory marker. Moreover, despite the statistical significance, the discriminative power of SIRI in terms of AUC value was not particularly high, and the study population was overall small. Accordingly, further studies with large samples and prospective designs are needed to validate these findings and comprehensively assess additional variables, such as the status of collaterals, brain reserve, infarct volume, and the occurrence of early complications as hemorrhagic transformation, which could be influencing the association between the SIRI and futile reperfusion [42].

## 5. Conclusions

Clinical outcome after EVT in patients with proximal anterior circulation AIS is often disappointing despite successful recanalization. Successful recanalization in patients with stroke does not necessarily translate into clinical improvement as recanalization is not equivalent to reperfusion, and reperfusion itself can cause injury in the brain [29]. This study revealed the SIRI as a predictor of futile endovascular reperfusion. Further understanding of the determinants and mechanisms underlying futile reperfusion may help to identify new therapeutic targets and develop strategies to improve the outcome of patients with acute ischemic stroke.

## Figures and Tables

**Table 1:** Baseline characteristics of patients.

	Futile Recanalization		*p*-Value
	No (*n* = 74)	Yes (*n* = 110)	
**Demographics**			
Age (years)	64 [53–76]	79 [69–83]	<0.001 ^a^
Male sex	39 (52.7)	48 (43.6)	0.227 ^b^
**Clinical history**			
Current smoking	17 (23.0)	22 (20.0)	0.629 ^b^
Hypertension	38 (51.4)	79 (71.8)	0.005 ^b^
Diabetes mellitus	6 (8.1)	17 (15.5)	0.140 ^b^
Dyslipidemia	36 (48.7)	48 (43.6)	0.503 ^b^
Coronary artery disease	17 (23.0)	17 (15.5)	0.198 ^b^
**Clinical assessment at admission**			
Systolic BP (mmHg)	134.9 (20.9)	140.3 (19.6)	0.076 ^c^
Diastolic BP (mmHg)	75.9 (11.3)	78.8 (12.5)	0.112 ^c^
NIHSS score	15 [10–18]	16 [13–18]	0.011 ^a^
ASPECT value	9 [7–10]	8 [7–10]	0.939 ^a^
Location of intracranial occlusion			0.349 ^b^
Internal carotid artery	13 (17.8)	16 (14.6)	
* Internal carotid artery terminus	1 (1.4)	5 (4.6)	
Middle cerebral artery			
M1 segment	51 (68.9)	68 (61.8)	
M2 segment	9 (12.2)	21 (19.1)	
Serum glucose (mg/dL)	110 [87–131]	116 [99–140]	0.071 ^a^
White blood cell count (×109/L)	9310 [7360–12,020]	9985 [7980–12,170]	0.244 ^a^
Absolute neutrophil count (×109/L)	6910 [5050–9500]	8040 [5990–10,140]	0.071 ^a^
Absolute monocyte count (×109/L)	590 [430–800]	675 [510–870]	0.066 ^a^
Absolute lymphocyte count (×109/L)	1325 [970–1970]	1130 [750–1580]	0.012 ^a^
SIRI (×109/L)	3.3 [1.6–5.3]	4.6 [2.4–8.3]	<0.001 ^a^
**Treatment**			
Intravenous thrombolysis	50 (67.6)	60 (54.6)	0.077 ^b^
Mechanical thrombectomy			0.606 ^b^
Stent-retrievers	38 (51.4)	60 (54.5)	
Aspiration catheters	25 (33.8)	30 (27.3)	
Both	11 (14.9)	20 (18.2)	

Data are presented as mean (SD) or median [IQR] for continuous variables and *n* (%) for categorical variables. * Associated internal carotid artery and middle cerebral artery occlusion (tandem occlusion). ^a^ Mann–Whitney test. ^b^ Chi-squared test. ^c^ Two-sample *t*-test. Abbreviations: ASPECT—Alberta Stroke Program Early CT, BP—blood pressure, IQR—interquartile range, NIHSS—National Institutes of Health Stroke Scale, SD—standard deviation, SIRI—systemic inflammation response index.

**Table 2:** Association between baseline characteristics and futile recanalization.

Dependent Variable	* Adjusted OR (95% CI)	*p*-Value
Age	1.07 (1.04–1.10)	<0.001
Male sex	0.85 (0.42–1.73)	0.661
History of hypertension	1.28 (0.62–2.67)	0.507
Baseline NIHSS score	1.10 (1.02–1.19)	0.013
ASPECT value	1.02 (0.80–1.30)	0.871
Admission SIRI	1.08 (1.01–1.17)	0.028

ORs for every 1-point increase in age, NIHSS score, ASPECT value, SIRI and for male sex and positive history of hypertension are obtained with logistic regression analysis. * Adjustment for age, sex, history of hypertension, baseline NIHSS score, ASPECT value, and admission SIRI. Abbreviations: ASPECT—Alberta Stroke Program Early CT, CI —confidence interval, NIHSS—National Institutes of Health Stroke Scale, OR—odds ratio, SIRI—systemic inflammation response index.

## Data Availability

Anonymized data will be shared by request from any qualified investigator.
